# Willingness to participate in combination screening for lung cancer, chronic obstructive pulmonary disease and cardiovascular disease in four European countries

**DOI:** 10.1007/s00330-023-10474-w

**Published:** 2023-12-07

**Authors:** Carina Behr, Hendrik Koffijberg, Maarten IJzerman, Hans-Ulrich Kauczor, Marie-Pierre Revel, Mario Silva, Oyunbileg von Stackelberg, Janine van Til, Rozemarijn Vliegenthart

**Affiliations:** 1 Health Technology and Services Research, Faculty of Behavioural and Management Science, University of Twente, Drienerlolaan 5, 7522 NB Enschede, The Netherlands; 2 Cancer Health Services Research, Centre for Health Policy, Melbourne School of Population and Global Health, Faculty of Medicine, Dentistry and Health Sciences, The University of Melbourne, Parkville, Melbourne, VIC 3010 Australia; 3 Erasmus School of Health Policy & Management, Rotterdam, The Netherlands; 4 Department of Diagnostic and Interventional Radiology, Heidelberg University Hospital, Im Neuenheimer Feld 110, 69120 Heidelberg, Germany; 5 Translational Lung Research Center, Member of the German Lung Research Center, Heidelberg, Germany; 6 Service de radiologie, Université de Paris, Assistance Publique des hôpitaux de Paris, Hôpital Cochin, 85 boulevard Saint-Germain, 75006 Paris, France; 7 Inserm U1016, Institut Cochin, 22 rue Méchain, 75014 Paris, France; 8 Scienze Radiologiche, Department of Medicine and Surgery (DiMeC), University of Parma, Pad. Barbieri, Ospedale Universitario di Parma, Via Gramsci 14, 43126 Parma, Italy; 9 Department of Radiology, University of Groningen, University Medical Centre Groningen, Hanzeplein 1, 9713 GZ Groningen, The Netherlands

**Keywords:** Patient preference, Mass screening, Lung neoplasms, Pulmonary Disease (Chronic Obstructive), Cardiovascular diseases

## Abstract

**Objectives:**

Lung cancer screening (LCS), using low-dose computed tomography (LDCT), can be more efficient by simultaneously screening for chronic obstructive pulmonary disease (COPD) and cardiovascular disease (CVD), the Big-3 diseases. This study aimed to determine the willingness to participate in (combinations of) Big-3 screening in four European countries and the relative importance of amendable participation barriers.

**Methods:**

An online cross-sectional survey aimed at (former) smokers aged 50–75 years elicited the willingness of individuals to participate in Big-3 screening and used analytical hierarchy processing (AHP) to determine the importance of participation barriers.

**Results:**

Respondents were from France (*n* = 391), Germany (*n *= 338), Italy (*n *= 399), and the Netherlands (*n *= 342), and consisted of 51.2% men. The willingness to participate in screening was marginally influenced by the diseases screened for (maximum difference of 3.1%, for Big-3 screening (73.4%) vs. lung cancer and COPD screening (70.3%)) and by country (maximum difference of 3.7%, between France (68.5%) and the Netherlands (72.3%)). The largest effect on willingness to participate was personal perceived risk of lung cancer. The most important barriers were the *missed cases* during screening (weight 0.19) and *frequency of screening* (weight 0.14), while *diseases screened for* (weight 0.11) ranked low.

**Conclusions:**

The difference in willingness to participate in LCS showed marginal increase with inclusion of more diseases and limited variation between countries. A marginal increase in participation might result in a marginal additional benefit of Big-3 screening. The amendable participation barriers are similar to previous studies, and the new criterion, *diseases screened for*, is relatively unimportant.

**Clinical relevance statement:**

Adding diseases to combination screening modestly improves participation, driven by personal perceived risk. These findings guide program design and campaigns for lung cancer and Big-3 screening. Benefits of Big-3 screening lie in long-term health and economic impact, not participation increase.

**Key Points:**

*• It is unknown whether or how combination screening might affect participation.*

*• The addition of chronic obstructive pulmonary disease and cardiovascular disease to lung cancer screening resulted in a marginal increase in willingness to participate.*

*• The primary determinant influencing individuals' engagement in such programs is their personal perceived risk of the disease.*

**Supplementary Information:**

The online version contains supplementary material available at 10.1007/s00330-023-10474-w.

## Objectives

Low-dose computed tomography (LDCT) lung cancer screening (LCS) can reduce mortality as reported after trials in the USA, the Netherlands, Germany and Italy [1–4]. In the USA and the UK, formal LCS programs have started [5, 6]. The EU’s recent publication, “A new approach” to cancer screening, highlights plans to incorporate LCS programs pending successful implementation trials [7]. A recent publication summarises the European progress and challenges of LCS implementation and identifies participation rates and health interventions such as smoking cessation, detecting chronic obstructive pulmonary disease (COPD) and comorbidities (cardiovascular disease (CVD)) [8].

High participation rates are essential to successful screening programs. Yet, the USA reports extremely low participation for LCS (7.3% of eligible smokers), while a cost-effective screening program requires at least 40% participation [8]. Therefore, during development, it is crucial to understand decision criteria of potential participants.

The potential added value of screening for multiple diseases is another opportunity to develop an efficient screening program. The ERS/ESR statement [9] highlights the value of focusing screening on the Big-3 killers, i.e. lung cancer, COPD (emphysema, bronchitis) and CVD (atherosclerosis), expected to cause the most deaths by 2050 [10]. Some attention has been drawn to the evidence of detecting the Big-3; however, the clinical effectiveness is not yet clear [11]. COPD screening within LCS may be valuable, due to a strong association between emphysema and risk of lung cancer, which may impact the follow-up of high-risk individuals [12–14]. The cost-effectiveness of coronary artery calcium scoring has been investigated in several reports with positive outcomes [15–19]; however, the evidence on COPD screening cost-effectiveness is very limited. In an early health technology assessment, the cost-effectiveness potential of Big-3 screening was shown, especially with the addition of CVD screening to LCS [20]. However, it is important to understand how combination screening influences potential participants’ decision to participate, as there will be additional risks and benefits (e.g. overdiagnosis and CVD event prevention). The extent of these risks and benefits is not yet known [11].

An established screening program shows that the crucial screening participation rates vary between countries. Breast cancer screening participation within Europe ranged from 21 to 82% in 2017 [21]. Except for reported screening trial participation rates, the expected LCS participation rates in different EU countries are currently unknown.

Previous research identified criteria influencing LCS participation [22–28]. The identified process and outcome criteria from literature (with examples) included location (≤10 min travel or the closest hospital from home), mode (scan or blood marker), accuracy (sensitivity, specificity or overdiagnosis), waiting time for result, radiation, anxiety, benefits of screening (reduced mortality and personal reassurance) and personal cost. Individual characteristics that were indicators of participation included sex, age, smoking status, lung function, motivation for enrolment (personal initiative, family or medical advice), distance to the referral centre, family history, education, body mass index, intention to quit smoking, social vulnerability and socio-professional category [22–28]. Some studies examined the relative importance of criteria. For instance, Broekhuizen et al [22] found location as more important in the Netherlands than accuracy and waiting time. See et al [23] identified early detection as the primary driver of participation.

This article assesses the impact of simultaneous screening for lung cancer, COPD and CVD on the willingness to participate amongst high-risk individuals in four European countries (France, Germany, Italy and the Netherlands). Additionally, the importance of screening decision criteria is assessed to inform effective implementation.

## Methods

The University of Twente gave ethical clearance (registration 210899). All respondents gave online informed consent before starting the survey.

An online cross-sectional survey targeted individuals aged 50–75 years without a lung cancer diagnosis who consider themselves current or former smokers. This cohort follows less strict inclusion criteria than screening trials. Ineligible respondents were identified through introductory questions (age and smoking history) and excluded from the survey. The survey targeted individuals from France, Germany, Italy and the Netherlands and was translated into the respective languages. The full survey is in the Supplementary Material.

### Survey design

The descriptive framework followed an iterative process, starting with the preference elicitation method, followed by the decision criteria. Preferences were elicited using analytic hierarchy process (AHP). AHP, a widely used multi-criteria decision analysis (MCDA) technique, aids preference-sensitive decisions involving multiple criteria. It supports individual or group decision-makers, revealing their own and stakeholders’ preferences [29]. Decision criteria are defined, and pairwise comparisons made, resulting in preference weights [30].

To elicit the willingness of respondents to participate in screening and answer the first research question, direct questions were used. Participants were asked what the likelihood is that they would participate in specific screening programs.

All respondents answered general questions on age, sex, educational level and smoking status, as well as family history for each of the Big-3 diseases. Following smoking status, questions were asked to estimate the pack-years smoked and, if applicable, how many years they have quit smoking. Other general risk-related questions included chest complaints for which they have not seen a physician, perceived 5-year risk of lung cancer, likelihood of smoking cessation within one year and likelihood of smoking cessation if diagnosed with one of the diseases.

### Criteria

Literature-sourced criteria driving screening participation were identified. Discussing key criteria led to two new criteria, resulting in a final list of eight. These criteria are relevant, non-redundant, non-overlapping and independent. The two added criteria were *Diseases screened for* and *immediate feedback.* The first is added due to this study’s focus on multi-disease screening. The second is adapted from mammography screening research, where results within 48 h are considered important [31]. Table 1 describes and provides ranges of the final eight criteria.

**Table 1:** Criteria influencing the decision to participate in screening

Criteria name	Description
*Location of screening*	Where the screening test will take place. You will have to travel to the screening location at your own cost. The location of screening can range between 10 min of travel from your home up to the closest hospital.
*Waiting time*	How long do you have to wait between taking the test and receiving the results? This can take between 1 and 7 days and might cause anxiety.
*Immediate feedback*	Will you get immediate feedback after your scan? Feedback could be given by a nurse, a radiologist or there could be no feedback at all. Feedback is not yet results.
*Number of screenings per 5-year period*	How often do you need to undergo the screening test? Depending on the diseases you are being screened for and the testing capacity of your country, this might vary. Possibilities are anything between yearly and every 5 years.
*Benefits of screening*	Screening programs are only introduced when they result in benefits such as fewer people dying from a certain disease or people living longer due to screening and early treatment. Benefits per 1000 screened individuals can be 3–5 averted deaths or 15 years in good health gained in total.
*Missed cases*	Screening tests do not always find all patients with a disease. There is always a small chance that after screening you are informed that you do not have the disease, when in fact it was just a missed case. You will then only be diagnosed in the next round of screening or when you seek medical attention due to symptoms. The number of missed cases can vary between 50 cases out of 1000 individuals for a severe disease and 100 cases out of 1000 individuals for a less severe disease.
*Follow-up tests*	Because everyone is different, screening tests sometimes indicate that there is something suspicious when in reality it is not the disease being screened for and nothing to be concerned about. In these cases, you will have to go for a follow-up test that might be a scan or invasive (where they cut out tissue to do some tests). This could cause anxiety. The number of follow-up tests per 1000 screened individuals can vary between 20 and 70 tests of which 1 is invasive.
*Diseases screened for*	Screening can be done to detect different diseases. In this study, we are considering screening for combinations of lung cancer, emphysema (a lung disease causing breathing difficulty) and coronary heart disease (calcium build-up in your heart which could cause events like a heart attack). No additional tests are needed when screening for more than one disease.
	Sub criteria for *diseases screened for* lung cancer screening; lung cancer and COPD screening; lung cancer and CVD screening; lung cancer, COPD and CVD screening.

*COPD* chronic obstructive pulmonary disease, *CVD* cardiovascular disease

**Fig. 1:**
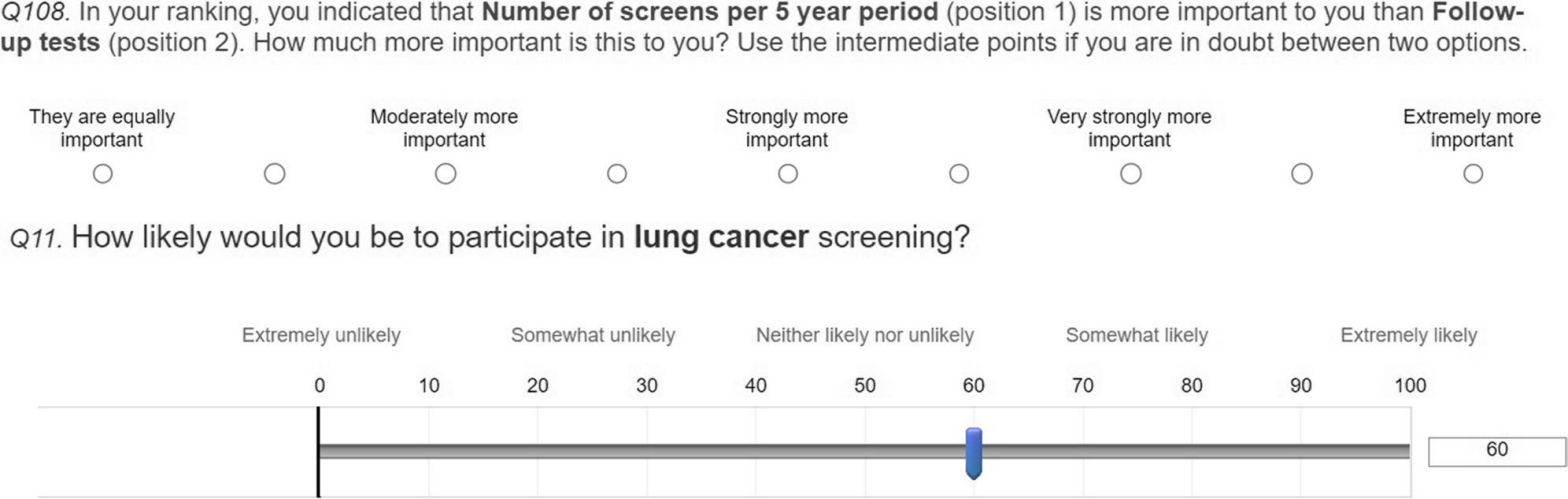
Example of a preference question and a stated willingness to participate question following the ranking of criteria in the English version of the survey. The full survey is available in the Supplementary Material

The preference elicitation section started with a straightforward ranking of the eight criteria, from most to least important. Then, respondents were asked to complete pairwise comparisons using a Likert scale to compare the relative importance of the criteria. Figure 1 shows an example of one of these questions. Only the top 5 criteria from the ranking were used in the pairwise comparisons, which reduced the number of comparisons from 28 to 10. The sub-criteria of *diseases screened for* (different combinations of Big-3 diseases) were also compared using pairwise comparisons. For the elicitation of preferences, only answers with a consistency ratio smaller than 0.3 (indicating the most consistent answers) were included. Due to the reduction in pairwise comparisons, the applied consistency ratio is at the upper limit of the typically accepted ratios of 0.1 to 0.3.

### Willingness to participate

To elicit respondents’ willingness to participate in screening, respondents were given more information on the benefits and risks of screening for each of the Big-3 diseases and asked what their likelihood of participation would be for screening for each of the combinations of diseases used as sub-criteria, as shown in Fig. 1. The respondent’s own answer to the likelihood of participating in LCS was provided in the following questions as a reference.

### Pilot testing

The translated surveys were used in think-aloud interviews to make improvements. Researchers from each country identified 5 eligible respondents to interview, using convenience sampling. A delay in ethical approval in Germany resulted in the survey being filled in by 5 researchers who were asked to imagine that they are part of the target group. The interviewees had to explain their thoughts when answering the survey to ensure that concepts were well understood. The guidelines used by interviewers can be found in the Supplementary Material.

After these interviews, translations of concepts were improved, and the option of indicating that a person smokes “occasionally” was added to be able to more accurately calculate the pack-years.

### Online survey

The online survey was set up in Qualtrics [32] and sent out by Dynata, a global online market research firm (https://www.dynata.com) between 1 and 28 December 2021. Dynata used a multi-sourcing panel recruitment model with a variety of contact methods including loyalty partnerships, apps and emails amongst others, which is a form of convenience sampling. In each country, a target of 330 respondents was set and recruitment was stopped after the quota was reached, which means that some participants were already recruited and could continue filling in the survey. Quotas were implemented to match population demographics per country.

### Statistical analysis

The statistical analysis was conducted in R version 4.2.2 [33]. Aggregation of individual priorities (AIP) with geometric mean was used to report the overall opinion of respondents [34]. The statistical significance (*p*<0.05) of differences in willingness to participate between subgroups was tested using the Kruskal-Wallis test. Furthermore, pairwise comparisons using the Wilcoxon rank sum test, with continuity correction data, were used to determine the statistical significance between groups.

The analysis could have confounding factors, influencing the factors with significant impact on the willingness to participate in screening. Therefore, we used a multivariate regression model to identify the factors that have a significant influence (*p*<0.05) on the willingness to participate while also correcting for the influence of other factors. This was used to confirm the importance of different factors on the willingness to participate in screening. The factors included in the multivariate regression to investigate their effect were age, smoking status, presence of already diagnosed COPD or CVD, sex, whether the respondent already had chest complaints, whether the respondent would plan to quit smoking within 1 year (if diagnosed with one of the Big-3), country, perceived 5-year lung cancer risk, education, pack-years and family history of each of the Big-3. Additionally, the interaction effects between the following factors were included: smoking status and the presence of diagnosed COPD or CVD, smoking status and the perceived lung cancer risk, smoking status and education level as well as pack-years and age or educational level.

## Results

The entire survey was filled in by 1470 respondents, 51.2% men, median age category 55–60 years, with a mean of 25 pack-years, with demographics shown in Table 2. The surveys were from France (*n* = 391), Germany (*n *= 338), Italy (*n *= 399), and the Netherlands (*n *= 342). Respondents with previously diagnosed COPD and CVD were 7% and 4% respectively, and less than 1% were diagnosed with both diseases.

**Table 2:** Demographics of eligible respondents that completed the survey

Demographics	Participants (*n*= 1470)
Age	
50–55	409 (27.8%)
55–60	332 (22.6%)
60–65	260 (17.7%)
65–70	241 (16.4%)
70–75	228 (15.5%)
Sex	
Men	753 (51.2%)
Women	714 (48.6%)
Non-binary	3 (0.2%)
Smoking status	
Current	786 (53.5%)
Former	564 (38.4%)
Occasional	120 (8.2%)
Education	
Lower secondary school or less	193 (13.1%)
Vocational school	376 (25.6%)
High school	394 (26.8%)
Bachelor’s	242 (16.5%)
Master’s	209 (14.2%)
PhD	43 (2.9%)
Missing	13 (0.7%)

*n* number, *PhD* Doctor of Philosophy

### Willingness to participate in screening

Table 3 shows the average willingness to participate in screening per disease and country, as well as the incremental willingness to participate in a specific screening program compared to LCS. The incremental value is an indication of the perceived added value of screening for additional diseases. The average varied between 70.3% for LC+COPD and 73.4% for the Big-3. There was a statistically significant difference in average willingness to participate between the different combinations of diseases (*p *< 0.001). In all countries, there was a higher willingness to participate in Big-3 screening compared to LCS alone (*p *= 0.006) and lung cancer with COPD (*p *< 0.001) screening, respectively. Additionally, the willingness to participate in screening for lung cancer and CVD was significantly higher compared to lung cancer and COPD (*p *= 0.02). In all countries, the willingness to participate in screening for lung cancer and COPD was the lowest. The willingness to participate in screening was significantly higher in the Netherlands than in France for any combination of diseases (*p *= 0.03).

**Table 3:** Average, standard deviation and incremental willingness to participate in screening per disease combination and country

	Average (standard deviation)				Incremental willingness to participate compared to LCS		
	LC	LC+COPD	LC+CVD	Big-3	LC+ COPD	LC+ CVD	Big-3
Netherlands (*n *= 432)	72.3 (25.4)	71.0 (25.9)	74.5 (24.6)	74.6 (24.9)	−1.3	2.2	2.6
Germany (*n *= 338)	71.6 (25.2)	71.3 (25.1)	73.2 (25.3)	74.7 (25.8)	−0.3	1.6	3.1
Italy (*n *= 399)	72.5 (22.3)	71.0 (22.5)	72.9 (22.2)	73.2 (22.6)	−1.5	0.4	0.7
France (*n *= 391)	68.5 (23.5)	68.0 (23.9)	69.4 (23.6)	71.7 (23.9)	−0.5	0.9	3.2
Overall	71.2 (24.1)	70.3 (24.3)	72.4 (23.9)	73.4 (24.3)	−0.9	1.2	2.2

*LC* lung cancer, *COPD* chronic obstructive pulmonary disease, *CVD* cardiovascular disease

Table 4 shows the willingness of respondents to participate in screening for different diseases based on their smoking status, personal perceived risk of developing lung cancer in the next 5 years and educational level. Current daily smokers were more willing to participate in LCS and lung cancer with COPD screening than former smokers (*p *< 0.003). The strongest relationship was with personal perceived risk, which increased for all screening combinations, from 69.2 to 82.7% for Big-3 screening (*p *< 0.0001). Finally, there seemed to be an inconsistent and not always statistically significant relationship between the educational level of respondents and their willingness to participate in screening, as respondents with both higher (Master’s) and lower (lower secondary school) levels of education were less willing to participate.

**Table 4:** Average and standard deviation of the willingness to participate in screening per disease combination for multiple factors

	LC	LC+COPD	LC+CVD	Big-3
Smoking status				
Current (*n *= 781)	73.0 (23.1)	72.2 (23.0)	72.9 (23.1)	73.9 (23.8)
Former (*n *= 559)	69.0 (24.6)	67.7 (25.2)	71.4 (24.6)	72.2 (24.9)
Occasional (*n *= 119)	69.7 (26.6)	69.9 (26.5)	73.5 (26.3)	76.0 (24.2)
Perceived risk of developing lung cancer in the next 5 years				
Low risk (*n *= 630)	65.8 (25.4)	65.0 (25.7)	67.7 (25.4)	69.2 (25.2)
Medium risk (*n *= 745)	74.6 (22.2)	73.4 (22.3)	75.4 (22.2)	76.0 (24.9)
High risk (*n *= 82)	81.5 (21.3)	80.7 (21.0)	80.7 (21.4)	82.7 (21.0)
Educational level				
Lower secondary school or less (*n *= 193)	68.4 (23.4)	67.8 (23.6)	68.8 (24.3)	70.1 (23.5)
Vocational school (*n *= 376)	72.1 (21.6)	71.2 (21.9)	73.6 (22.6)	74.3 (22.9)
High school (*n *= 394)	72.2 (25.1)	70.6 (25.0)	73.0 (24.7)	73.3 (25.0)
Bachelor’s (*n *= 242)	71.2 (23.8)	71.0 (24.4)	73.1 (23.6)	74.4 (24.2)
Master’s (*n *= 209)	68.9 (23.0)	68.0 (23.9)	70.8 (23.0)	73.1 (23.0)
PhD (*n *= 43)	76.5 (24.7)	76.0 (24.2)	77.0 (24.0)	79.3 (25.3)

*LC* lung cancer, *COPD* chronic obstructive pulmonary disease, *CVD* cardiovascular disease, *PhD* Doctor of Philosophy

There was no significant difference between the willingness of respondents to participate in screening based on age, sex or pack-years. Respondents with a family history of lung cancer had a 5% higher willingness to participate in screening than those without and so did those with a family history of COPD (5% increase) and CVD (8% increase). The differences in willingness to participate between all diseases for respondents who ranked “Diseases screened for” in their top four criteria were the same as those who ranked this criterion amongst the four least important. The multivariate regression confirmed the importance of the univariate effects of the factors presented in Tables 3 and 4 when correcting for other variables.

### Relative importance of decision criteria for participation

Of the 1470 respondents, 1127 (76.7%) answered the pairwise comparisons consistently. When comparing the eight decision criteria, the most important criteria related to willingness to participate in screening were the *number of cases missed* during screening (weight 0.19, standard deviation (SD) = 0.042) and the *frequency of screening* (weight 0.14, SD = 0.050). These criteria were followed by *waiting time* (0.13, SD = 0.050), *follow-up tests* (0.12, SD = 0.047), *benefits of screening* (0.12, SD = 0.074), *location of screening* (0.11, SD = 0.067), *diseases screened for* (0.11, SD = 0.074) and *immediate feedback on the scan* (0.09, SD = 0.059). The order of importance of criteria did not change when removing inconsistent results, and the weights of criteria changed by at most 0.013.

## Conclusion

The results show a relatively high willingness to participate in screening for all Big-3 diseases on chest LDCT with marginal differences between countries. There was a marginal but statistically significant difference in willingness to participate in screening for the Big-3 diseases compared to either LCS alone or in combination with either COPD or CVD. One of the strongest factors related to willingness to participate in screening was the perceived lung cancer risk of respondents.

Interestingly, while the overall willingness to participate in screening is high, it is lower for lung cancer with COPD screening than LCS alone. This may be because of greater awareness of CVD (events) burden than COPD burden [35]. It could also be influenced by the stigma around COPD or by the information presented to respondents, which highlights smoking cessation as the most effective intervention for COPD management [36]. Nevertheless, adding COPD to combined lung cancer and CVD screening did increase the willingness to participate.

The stated willingness to participate in screening was relatively high and only marginally different between the countries. As the screening programs considered in this study are not currently available in these countries, results could not be compared to actual screening participation. However, the difference in participation rates for existing screening programs is much larger. Participation in breast cancer screening in 2019 ranged from 48.8% in France to 76.1% in the Netherlands [37]. Participation in the Netherlands is very close to the stated preference in this study, while the French revealed participation is much lower. It could indicate that respondents have much higher stated participation than actual participation, or that French interest in participation in LCS is much higher than in breast cancer screening. Our survey results suggest that expected participation rates in France, Germany, Italy and the Netherlands would not differ significantly if the same marketing, approach and organisation of screening is provided.

The factor contributing to the largest increase in an individual’s willingness to participate in screening was a high perceived risk of lung cancer, which corroborates previous research findings [38]. Other similarities include that individuals with COPD and other respiratory diseases [39, 40] are more likely to participate in screening and that the expected participation rate is very high in Western Europe (83.6% in Belgium) [41]. Differences include studies that found men [39], older individuals [38] and individuals with a high level of education [40] more likely to participate in screening; however, these studies were conducted in other countries.

Although there was a statistically significant difference between respondents’ willingness to participate in screening for different diseases, it is currently unknown how a difference of at most 3.1% might impact the (cost-)effectiveness of chest LDCT screening programs. The impact of participation rates on the cost-effectiveness of LCS has been investigated in previous studies. Two studies concluded that a decreased participation rate decreases the cost-effectiveness ratio of screening [42, 43] without a clear explanation, while two other studies found no difference in the cost-effectiveness ratio with changing participation rate, as costs and effects change proportionally [44, 45]. These studies did not consider changing participation rates in specific target populations. In colorectal cancer screening in Australia, a 10% increase in participation was associated with an additional 24,300 prevented cancer cases and 24,800 deaths prevented, which is an additional 28% of deaths prevented [46]. Cost-effectiveness and the survival benefit of LDCT screening could improve with increased participation in specific subgroups, such as current smokers, assuming that the long-term health gains in this group would be larger than in former smokers. However, further research is needed to confirm this hypothesis.

High diagnostic sensitivity (i.e. few missed cases) and screening frequency (i.e. achieving health benefits with a low screening frequency) were the most important decision criteria for respondents. The relative importance of decision criteria was consistent with previous research. The criteria added for this study (*diseases screened for and immediate feedback*) were not perceived as very important compared to other decision criteria.

Limitations are, firstly, that this study only elicited preferences of potential participants (according to current but less strict inclusion criteria), which will not necessarily correspond to the revealed preferences during implementation. In colon cancer screening, for example, it was found that actual participation in screening was much lower than the stated willingness to participate, elicited beforehand (40–50% vs 66–88%) [47]. Secondly, the respondents of this survey may already have had a propensity for participating in general (a form of self-selection bias). Thirdly, Dynata used a panel recruitment strategy, which is a form of convenience sampling. Finally, spirometry assessment would be a more accurate diagnostic tool for COPD; however, this study focuses on the extension of the already performed chest LDCT, by evaluating emphysema presence as a valuable addition.

In conclusion, the expected participation rates of Big-3 screening with LDCT are slightly higher than the high expected participation rate for LCS alone in the investigated European countries. Big-3 screening may therefore marginally increase screening benefits compared with LCS only depending on screening cost, diagnostic accuracy, treatment options, adherence, and benefits, for COPD and CVD.

## Supplementary Information

Below is the link to the electronic supplementary material.

Supplementary file1 (PDF 1057 KB)

## Abbreviations

AHP: Analytic hierarchy process
AIP: Aggregation of individual priorities
COPD: Chronic obstructive pulmonary disease
CVD: Cardiovascular disease
LCS: Lung cancer screening
LDCT: Low-dose computed tomography
MCDA: Multi-criteria decision analysis
SD: Standard deviation

## Declarations

### Guarantor

The scientific guarantor of this publication is Rozemarijn Vliegenthart.

### Statistics and biometry

No complex statistical methods were necessary for this paper.

### Methodology

• prospective

• online survey study

• multinational study (participants were not related to specific centres)
