# Supplementary material for: Willingness to participate in combination screening for lung cancer, chronic obstructive pulmonary disease and cardiovascular disease in four European countries
Source: Eur Radiol. 2023 Dec 7;34(7):4448–56. doi: 10.1007/s00330-023-10474-w (PMC11213747; doi:10.1007/s00330-023-10474-w)
Supplement: Supplementary file 1 — Supplementary file1 (PDF 1057 KB) [file 330_2023_10474_MOESM1_ESM.pdf]

Willingness to participate in combination screening for lung cancer, chronic obstructive pulmonary disease and cardiovascular disease in four European countries

Electronic Supplementary Material (ESM)

Eur Radiol (2023) Behr C, Koffijberg H, IJzerman M et al

Contents

Interview guidelines 1

English Survey 3

Introduction questions 4

AHP Reduction Questions 6

LDCT info 7

AHP 8

Disease preferences 11

LC background 13

LC and emphysema 14

LC and CHD 15

LC, emphysema and CHD 15

Concluding questions 16

Interview guidelines

Dear interviewer,

You will be conducting five think-aloud interviews with respondents aged 50-75 years old who are current and former smokers who have not been diagnosed with lung cancer. In these interviews, respondents fill in the online survey themselves and as they do this, they are asked to think-out-loud while responding to the survey. The goal of these interviews is to get feedback on our survey in different country settings to see if the survey is understandable by respondents from 4 countries each in their language. Following these interviews, some adjustments will be made to the survey and then the survey should be understandable and ready to be sent online to eligible respondents who will fill in the survey completely independently. The feedback we require of you is thus to ensure that even if you were not present in the interview, the respondents would be able to respond to the survey independently. Please read the following 6 points carefully before planning interviews with your respondents.

Thank you for your assistance in this project!

1. Identify respondents

Please identify 5 individuals from your country who are 50-75 years old, are current or former smokers and have not been diagnosed with lung cancer before. Make sure that you comply with the EU General Data Protection Regulation (GDPR), therefore do not use contact information from databases for which you have access. Rather identify individuals who are colleagues, friends or someone you know. Please make sure that not all respondents are highly qualified or familiar with screening.

2. Decide how you will conduct the interviews

You can choose how you want to conduct the survey. The survey will be filled in by the respondents online, but the respondent should be able to speak to you while filling in the survey. My suggestion is to use video-calling software, where you or the respondent have the survey open and share your screen. In this way, you can easily speak to the respondent and have them answer the survey questions. If the other person is not able to use video-calling software, I would suggest sending the respondent the link to the survey and have them fill it in on a PC or mobile phone while you are on a normal phone call with them. You can also conduct the interview in person, but please keep local Covid measures in mind and remember that we do not give reimbursement for individuals travelling to your location. It is not a problem if you decide on a different approach for each individual. Make

sure to discuss the mode of the interview with the respondent when making the appointment to ensure that they have the facilities to conduct a video call for example.

What is important is that the respondent can speak to you while they are filling in the survey.

3. Check translation

Please read through the original English and the translated version before you conduct the interviews. The translations have been done by professional native speakers, but there can always be small interpretation errors or jargon from the field which the translators might have missed. If you find a translation issue which you can solve yourself, please report this and indicate what you would like to change so it can be changed in the online version of the survey.

4. Before the interview

Because we do not want to create any bias on the respondents to have more information than online respondents, we want to limit the amount of information given before the interview. Before the interview mention to the respondent that you will help them fill in a survey which concerns their preferences on lung cancer screening and that no personally identifying data will be recorded. You can also mention that their response will not be used in the analysis, but is only used to improve the questions and understandability of the survey. We expect the interviews to take about 30-40 minutes (the survey without an interview takes approximately 12 minutes to fill in if the respondent reads all the material). You can answer any questions about how the interviews will be conducted, but not of the extended aim of the study. This can be explained after the interview. You can print the "Notes for individual interviews" on the last page of this document to make notes and to remind yourself what you need to look out for.

5. During the interview

Before starting the interview, explain to the respondent that no personally identifiable information will be recorded and sent to any other parties outside of the interview. Also, explain to the respondent how the interview will work. The respondent needs to show you how they fill in the survey and has to explain what they are thinking while answering the questions to indicate what kind of comparisons they are thinking of when responding. While conducting the interview, please be cautious of not explaining additional information to the respondents while they are filling in the survey. If they have a question, you can answer it and make a note of the question and your response, but they should mostly be filling in the survey by themselves and only explaining to you what they do not understand. You should however ask them what are they thinking with each choice they make in the survey if they are not doing this.

6. Make notes

Do not write down any personal information of the respondent such as name or date of birth. While doing the interviews, please make notes on what the respondents ask as well as what you responded to the respondent (e.g. which concepts they did not understand, response scales that were misunderstood or information they felt was missing). You can use page 4 as a guideline. Try to see if the respondent is thinking the way we expect them to (i.e. I think the one aspect is more important than the other because of my experience or because of the experience a friend had). You do not have to make notes of the respondent's answers, as this will be saved on Qualtrics who hosts the survey. We expect the interviews to take longer than a normal survey response, but please also write down how long the respondents took to fill in the survey from the first welcoming message until the completion message. If a respondent has a different likelihood of participation for screening for any combination of diseases compared to their likelihood of participation in lung cancer screening only, make a note of why this response changes.

7. Closing the interview

Thank the respondent for their time and willingness to voluntarily participate in the interview. Ask the respondent if they think the survey was set up in a neutral way (both benefits and risks of screening was indicated clearly for them to be able to make the tradeoff). Does the respondent have any other comments about information or options that were unclear or missing?

If the respondent is interested, you can answer any questions regarding the aim of the study which is: This study aims to determine eligible lung cancer screenees' willingness to participate in LDCT lung cancer screening in different combinations with emphysema and CHD as well as identifying the relative importance of decision criteria for participation. The study is being conducted in the Netherlands, Germany, Italy and France.

8. Report back

Please report back on an aggregated level in English. Report back what were the major and minor problems during the interviews. Mention missing information, questions asked by respondents, issues with translation and what the respondents found difficult to understand. Please also indicate if there were any issues with the survey itself (navigating through the survey, buttons that did not work etc.). Also, report back how long each respondent took to fill in the survey. You can use the table below as a reference frame.

Criteria	Feedback (summarised for all respondents)
Unclear concepts/ Was the survey neutral (risks vs benefits)	
Questions from respondents	
Issues with translation	
Technical issues with the survey	
Missing information	
Any other comments you think is important	
Time from welcoming message to thank you message (per respondent)	
Respondent 1	
Respondent 2	
Respondent 3	
Respondent 4	
Respondent 5	

Notes for individual interviews

Before the interview mention to the respondent that you will **help them fill in a survey** which concerns their preferences on lung cancer screening and that **no personally identifying data** will be recorded. You can also mention that their response will not be used in the analysis, but is only used to **improve the questions and understandability** of the survey. We expect the interviews to take about **30-40 minutes** (the survey without an interview takes approximately 12 minutes to fill in). You can answer any questions about how the interviews will be conducted, but not of the extended aim of the study. This can be **explained after the interview**.

Criteria	Feedback
Unclear concepts/ Was the survey neutral (risks vs benefits)	
Questions from respondents	
Issues with translation	
Technical issues with the survey	
Missing information	
Any other comments you think is important	
Time from welcoming message to thank you message (per respondent)	
Respondent	

Thank the respondent for their time and willingness to voluntarily participate in the interview. If the respondent is interested, you can answer any questions regarding the aim of the study which is: This study aims to determine eligible lung cancer screenees' willingness to participate in LDCT lung cancer screening in different combinations with emphysema and CHD as well as identifying the relative importance of decision criteria for participation. The study is being conducted in the Netherlands, Germany, Italy and France.

English Survey

Welcome

Dear participant,

You are being invited to participate in a survey titled “Citizens’ perspective oncombination screening”.

This survey aims to determine the preferences of a high-risk population for participation in screening for lung cancer with the potential addition of emphysema,coronary heart disease or both using low-dose CT.

This survey is being initiated by a team of researchers from the University of Twente in the Netherlands; University of Heidelberg, Germany; University of Parma, Italy andUniversité de Paris, France as part of the B3Care project.

We hope that you are willing to complete this survey, which will take approximately 10to 15 minutes. Your participation in this survey is entirely voluntary and you can withdraw at any time. By continuing to the next question you are giving informed consent.

We believe there are no risks associated with this research survey, however, information that will be provided about potential risks for having diseases may causedistress.
As with any online related activity, the risk of a data breach is always possible. We minimize any risks by storing data on secured servers, removing the survey and theacquired data from the server at the end of the study and by not capturing data thatcan be related to you as a person (IP address, e-mail address, traceable personal data such as date of birth).

Ethical clearance for this study was obtained from the University of Twente (210899).If you have any questions regarding this study or this survey, you can contact the coordinator, Carina Behr, at c.m.behr@utwente.nl

Thank you for considering to participate,
The research team

Introduction questions

What is your age?

[▾]

Do you smoke tobacco?

- ○ Yes
- ○ I used to
- ○ Occasionally
- ○ No

Have you been diagnosed with any of the following diseases?

- ○ Lung cancer/tumour

- ○ Emphysema/COPD
- ○ Coronary heart disease
- ○ None of the above

Subquestions for: Occasional smokers

Which one of the following applies to you?

- ○ I always only smoked occasionally
- ○ I smoked more often and changed to smoking only occasionally

How many years have you been smoking for? (rounded to a whole number)

How many years did you smoke for? (rounded to a whole number)

How many years ago did you stop smoking? (rounded to a whole number)

How many years did you smoke more than only occasionally? (rounded to a wholenumber)

How many years have you been smoking occasionally? (rounded to a whole number)

On average, how many cigarettes did you smoke per day?

On average, how many cigarettes do you smoke per day?

On average how many cigarettes do you currently smoke per month?

AHP Reduction Questions

Multiple factors might affect your willingness to participate in a screening program. Wehave some decision criteria listed and explained in the table below. After the table, you will be asked how important these are to you in your decision to participate in screening.

Location of screening	Where the screening test will take place. You will have to travel tothe screening location at your own cost. The location of screening can range between 10 minutes of travel from your home up to the closest hospital.
Waitingtime	How long you have to wait between taking the test and receiving the results. This can take between 1 and 7 days and might cause anxiety.
Immediate feedback	Will you get immediate feedback after your scan? Feedbackcould be given by a nurse, a radiologist or there could be nofeedback at all. Feedback is not results yet.
Number of screenings per 5 year period	How often do you need to undergo the screening test?<br>Depending on the diseases you are being screened for and thetesting capacity of your country, this might vary. Possibilities are anything between yearly or every five years.
Benefits of screening	Screening programs are only introduced when they result in benefits such as fewer people dying from a certain disease orpeople living longer due to screening and early treatment.<br>Benefits per 1,000 screened individuals can be 3-5 averted deaths or 15 years in good health gained in total.
Missedcases	Screening tests do not always find all patients with a disease. There is always a small chance that after screening you would think that you do not have the disease, when in fact it was just a missed case. You will then only be diagnosed in the next round ofscreening or when you seek medical attention due to symptoms. The number of missed cases can vary between 50 cases out of 1,000 individuals for a severe disease and 100 cases out of 1,000 individuals for a less severe disease.
Follow-uptests	Because everyone is different, screening tests sometimes indicate that there is something suspicious when in reality it is notthe disease being screened for and nothing to be concerned about. In these cases, you will have to go for a follow-up test thatmight be a scan or invasive (where they cut out tissue to do some tests). This could cause anxiety. The number of follow-up tests per 1,000 screened individuals can vary between 20 and 70tests of which 1 is invasive.
Diseases screened for	Screening can be done to detect different diseases. In this study,we are considering screening for combinations of lung cancer, emphysema (a lung disease, causing breathing difficulty) and coronary heart disease (calcium build up in your heart which could cause events like a heart attack). No additional tests are needed when screening for more than one disease.

Rank the following decision criteria from most (1) to least (8) important when you decide whether you would participate in lung cancer screening or not. Drag and drop the items to complete ranking.

Missed cases

Follow-up tests

Benefits of screening

Immediate feedback

Diseases screened for

Waiting time

Number of screens per 5 year period

Location of screening

LDCT info

Screening is the process in which high-risk individuals who do not have any symptoms, undergo a test to detect the disease as early as possible. A well-known example of screening is mammography to detect breast cancer. Detecting the diseaseearlier helps with effective treatment and could lead to patients living longer on average.

In this survey, we are only considering screening using Low-dose CT. This screeningmethod has a small radiation exposure and does not hurt. In a screening context, thescan will be done at your regional hospital, free of charge.

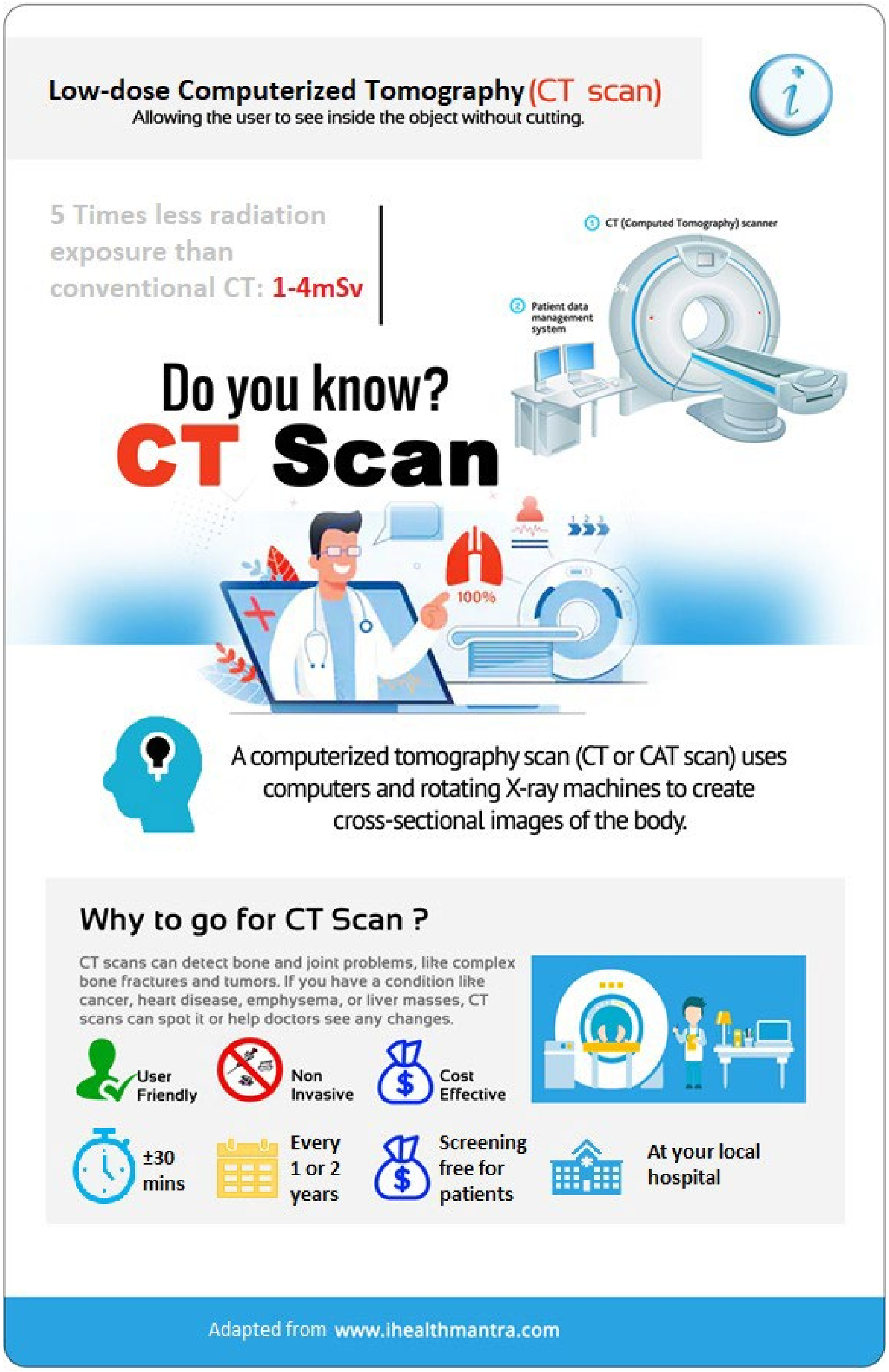

AHP

In your ranking, you indicated that **${e://Field/AHPRank1}** (position 1) is more important to you than **${e://Field/AHPRank2}** (position 2). How much more importantis this to you? Use the intermediate points if you are in doubt between two options.

They are equally important	Moderately more important	Strongly more important	Very strongly more important	Extremely more important

In your ranking, you indicated that **${e://Field/AHPRank1}** (position 1) is more important to you than **${e://Field/AHPRank3}** (position 3). How much more importantis this to you? Use the intermediate points if you are in doubt between two options.

They are equally important	Moderately more important	Strongly more important	Very strongly more important	Extremely more important

In your ranking, you indicated that **${e://Field/AHPRank1} (position 1)** is more important to you than **${e://Field/AHPRank4}** (position 4). How much more importantis this to you? Use the intermediate points if you are in doubt between two options.

They are equally important	Moderately more important	Strongly more important	Very strongly more important	Extremely more important

In your ranking, you indicated that **${e://Field/AHPRank1}** (position 1) is more important to you than **${e://Field/AHPRank5}** (position 5). How much more importantis this to you? Use the intermediate points if you are in doubt between two options.

They are equally important	Moderately more important	Strongly more important	Very strongly more important	Extremely more important

In your ranking, you indicated that **${e://Field/AHPRank2}** (position 2) is more important to you than **${e://Field/AHPRank5}** (position 5). How much more important is this to you? Use the intermediate points if you are in doubt between two options.

They are equally important	Moderately more important	Strongly more important	Very strongly more important	Extremely more important

○ ○ ○ ○ ○ ○ ○ ○ ○

In your ranking, you indicated that **${e://Field/AHPRank2}** (position 2) is more important to you than **${e://Field/AHPRank4}** (position 4). How much more importantis this to you? Use the intermediate points if you are in doubt between two options.

They are equally important	Moderately more important	Strongly more important	Very strongly more important	Extremely more important

○ ○ ○ ○ ○ ○ ○ ○ ○

In your ranking, you indicated that **${e://Field/AHPRank2}** (position 2) is more important to you than **${e://Field/AHPRank3}** (position 3). How much more importantis this to you? Use the intermediate points if you are in doubt between two options.

They are equally important	Moderately more important	Strongly more important	Very strongly more important	Extremely more important

○ ○ ○ ○ ○ ○ ○ ○ ○

They are equally important	Moderately more important	Strongly more important	Very strongly more important	Extremely more important

○ ○ ○ ○ ○ ○ ○ ○ ○

In your ranking, you indicated that **${e://Field/AHPRank3}** (position 3) is more important to you than **${e://Field/AHPRank4}** (position 4). How much more importantis this to you? Use the intermediate points if you are in doubt between two options.

They are equally important	Moderately more important	Strongly more important	Very strongly more important	Extremely more important

○ ○ ○ ○ ○ ○ ○ ○ ○

In your ranking, you indicated that **${e://Field/AHPRank3}** (position 3) is more important to you than **${e://Field/AHPRank5}** (position 5). How much more importantis this to you? Use the intermediate points if you are in doubt between two options.

They are equally important	Moderately more important	Strongly more important	Very strongly more important	Extremely more important

○ ○ ○ ○ ○ ○ ○ ○ ○

In your ranking, you indicated that **${e://Field/AHPRank4}** (position 4) is more important to you than **${e://Field/AHPRank5}** (position 5). How much more importantis this to you? Use the intermediate points if you are in doubt between two options.

They are equally important	Moderately more important	Strongly more important	Very strongly more important	Extremely more important

○ ○ ○ ○ ○ ○ ○ ○ ○

Disease preferences

This study considers screening for combinations of lung cancer, emphysema andcoronary heart disease. All three these diseases can be detected on a single test,meaning for more than one disease you would still only have to attend screening once. Shared risk factors of these three diseases are smoking and age.

Lung cancer is a malignant tumor in the lungs, where cells grow in an uncontrolledmanner. Lung cancer often causes coughing (of blood) and shortness of breath.
Emphysema (a type of COPD) is a disease in the lungs which causes difficulty tobreathe.
Coronary heart disease is caused by calcium build-up in the arteries of the heartwhich could cause a heart attack.

The following questions will be about your preference for screening for certaincombinations of these diseases.

If you have to undergo screening, which of the following options would you prefer?

- ○ Lung cancer screening only
- ○ Lung cancer screening with simultaneous emphysema screening
- ○ I do not have a preference

In the previous question you indicated that you prefer "${q://QID129/ChoiceGroup/SelectedChoices}". How strong is your preference for this screening option?

Moderately preferred	Strongly preferred	Very strongly preferred	Extremely preferred
○	○	○	○

If you have to undergo screening, which of the following options would you prefer?

- ○ Lung cancer screening only
- ○ Lung cancer screening with simultaneous coronary heart disease screening
- ○ I do not have a preference

In the previous question you indicated that you prefer "${q://QID131/ChoiceGroup/SelectedChoices}". How strong is your preference for thisscreening option?

Moderately preferred	Strongly preferred	Very strongly preferred	Extremely preferred
○	○	○	○

If you have to undergo screening, which of the following options would you prefer?

- ○ Lung cancer screening with simultaneous emphysema screening
- ○ Lung cancer screening with simultaneous coronary heart disease screening
- ○ I do not have a preference

In the previous question you indicated that you prefer "${q://QID133/ChoiceGroup/SelectedChoices}". How strong is your preference for thisscreening option?

Moderately more important	Strongly more important	Very strongly more important	Extremely more important
○	○	○	○

If you have to undergo screening, which of the following options would you prefer?

- ○ Simultaneous lung cancer, emphysema and coronary heart disease screening
- ○ Lung cancer screening with simultaneous coronary heart disease screening
- ○ I do not have a preference

In the previous question you indicated that you prefer "${q://QID135/ChoiceGroup/SelectedChoices}". How strong is your preference for this screening option?

Moderately more important	Strongly more important	Very strongly more important	Extremely more important
○	○	○	○

If you have to undergo screening, which of the following options would you prefer?

- ○ Lung cancer screening with simultaneous emphysema screening
- ○ Simultaneous lung cancer, emphysema and coronary heart disease screening
- ○ I do not have a preference

In the previous question you indicated that you prefer "${q://QID136/ChoiceGroup/SelectedChoices}". How strong is your preference for thisscreening option?

Moderately more important	Strongly more important	Very strongly more important	Extremely more important
○	○	○	○

LC background

Lung cancer screening can be combined with screening for emphysema and/or coronary heart disease. If these diseases are screened for in a combined screening program, it is possible that multiple diseases can be detected, but the chances of false detection or missed cases also rise. More information about each disease can be found in the following parts of the survey.

Did you know that if you screen 1000 individuals with a high risk of lung cancer in 3 screening rounds, 779 will have scans indicating that nothing is wrong, 180 will needto go for another test to make sure they don't have lung cancer (13 of these will be abiopsy) and 41 will be diagnosed with lung cancer early. Four of the diagnosed lung cancers would not have caused the person harm and 3 would not die of lung cancerthanks to screening). Look at the following picture to see what this means:

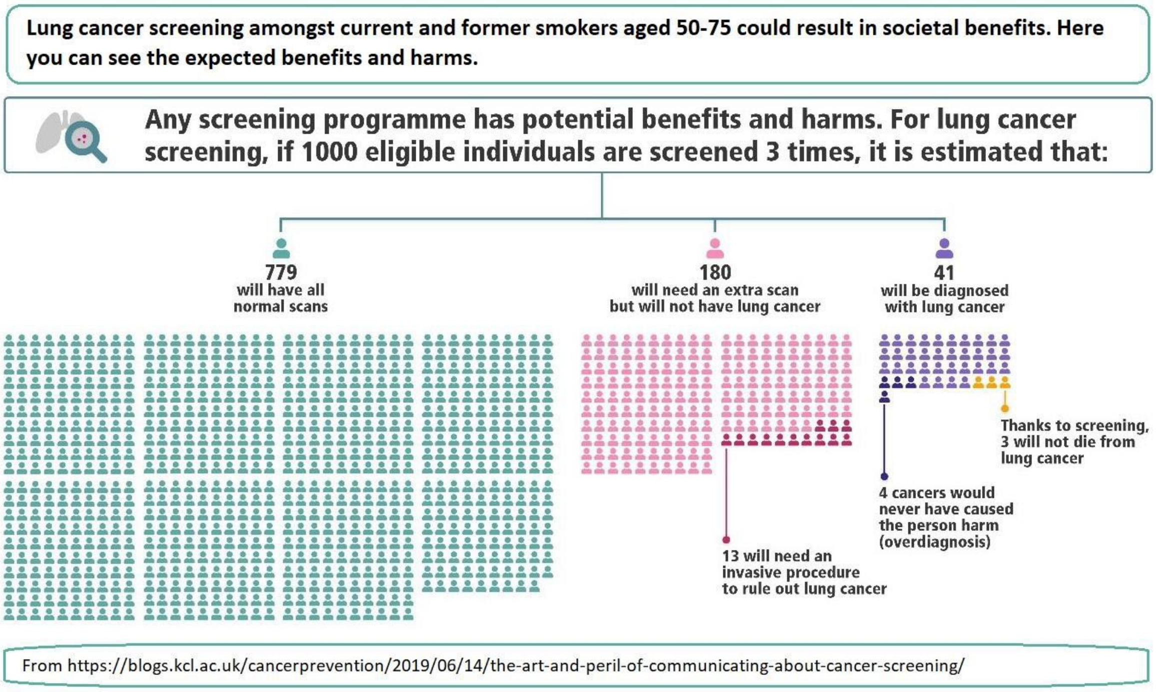

How likely would you be to participate in **lung cancer** screening?

Extremely unlikely	Somewhat unlikely	Neither likely nor unlikely	Somewhat likely	Extremely likely

0 10 20 30 40 50 60 70 80 90 100

LC and emphysema

Emphysema can be detected on the same low-dose CT as lung cancer. Emphysemais a disease of the lungs, which causes shortness of breath and gets worse with time.If you would screen for emphysema and lung cancer simultaneously, emphysema could also be detected, and symptoms could be treated early. This is a benefit of screening for emphysema. However, screening for emphysema too could also mean that you would increase the potential harms. You might need more tests if there is a suspicion that you have emphysema, and you could be diagnosed and receive treatment for emphysema if you don’t need it.

Did you know that if you screen 1000 individuals with a high risk of emphysema, 650will have scans indicating that nothing is wrong, 69 will need to go for another test tomake sure they don't have emphysema (32 of these will also use medication unnecessarily), and 281 will be assisted to try to slow down the progression of the disease. The diagnosed patient will be encourage to stop smoking. Look at the following picture to see what this means:

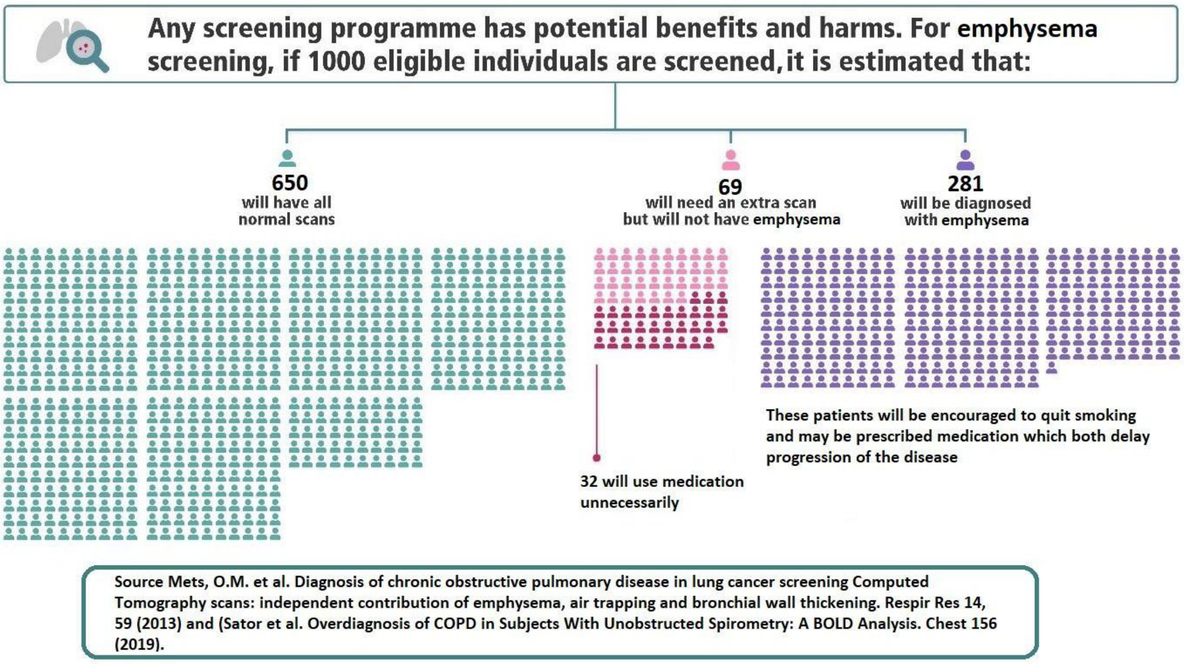

How likely would you be to participate in a screening program which simultaneouslyscreens for **lung cancer and emphysema**?

Use your answer for lung cancer screening-only (${q://QID1/ChoiceNumericEntryValue/1}) as reference

Extremely unlikely	Somewhat unlikely	Neither likely nor unlikely	Somewhat likely	Extremely likely

0 10 20 30 40 50 60 70 80 90 100

LC and CHD

Simultaneously screening for **Coronary heart disease (CHD)** could be beneficial when the disease(s) are detected and treated early. 5 individuals will not die of CHD if 1,000 individuals are screened, but there is also the risk of being diagnosed and treated when the heart disease would never have caused any damages.

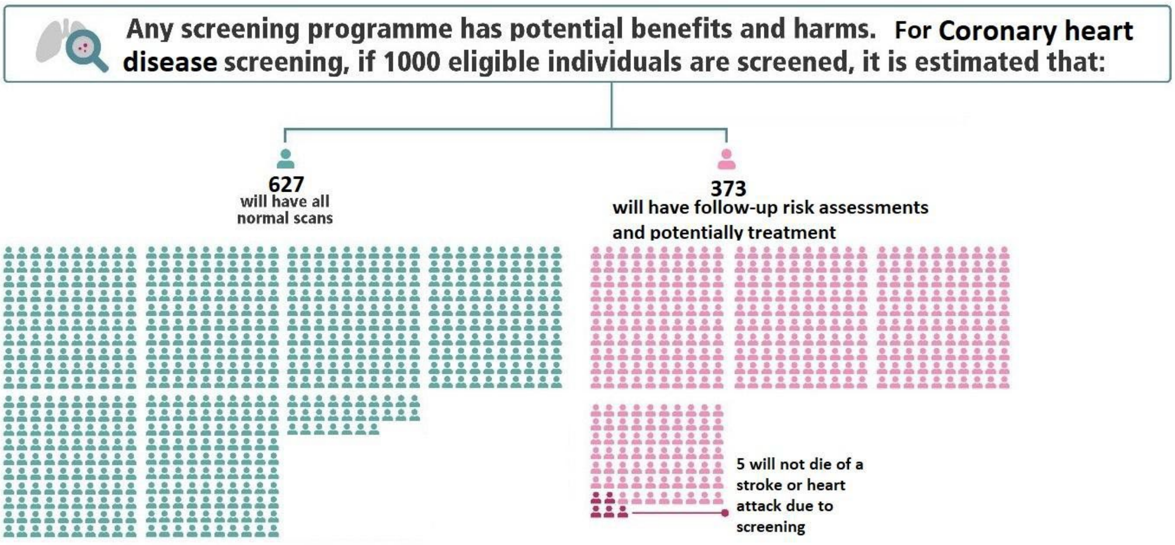

How likely would you be to participate in a screening program which simultaneously screens for **lung cancer** and **coronary heart disease**?

Use your answer for lung cancer screening-only (${q://QID1/ChoiceNumericEntryValue/1}) as reference

Extremely unlikely	Somewhat unlikely	Neither likely nor unlikely	Somewhat likely	Extremely likely

0 10 20 30 40 50 60 70 80 90 100

LC, emphysema and CHD

How likely would you be to participate in a screening program which simultaneouslyscreens for **lung cancer**, **emphysema** and **coronary heart disease**?

Use your answer for lung cancer screening-only (${q://QID1/ChoiceNumericEntryValue/1}) as reference

Extremely unlikely	Somewhat unlikely	Neither likely nor unlikely	Somewhat likely	Extremely likely

0 10 20 30 40 50 60 70 80 90 100

Concluding questions

What is your sex?

- ○ Male
- ○ Female
- ○ Non-binary

Do you have any chest complaints that you are aware of but will not lead you to see aphysician yet?

- ○ Yes
- ○ No

What do you think your risk is to get lung cancer in the next 5 years?

- ○ Low
- ○ Medium
- ○ High

What is your highest level of education?

- ○ Lower secondary school or less
- ○ Trade/vocational/professional school
- ○ High school graduate
- ○ Bachelor's degree
- ○ Master's degree
- ○ PhD

How likely are you to stop smoking in the next year?

- ○ Extremely likely
- ○ Somewhat likely
- ○ Neither likely nor unlikely
- ○ Somewhat unlikely
- ○ Extremely unlikely

How likely do you think you are to stop smoking if you would be diagnosed with lungcancer in the next year?

- ○ Extremely likely
- ○ Somewhat likely
- ○ Neither likely nor unlikely
- ○ Somewhat unlikely
- ○ Extremely unlikely

How likely do you think you are to stop smoking if you would be diagnosed withemphysema in the next year?

- ○ Extremely likely
- ○ Somewhat likely

- Neither likely nor unlikely
- Somewhat unlikely
- Extremely unlikely

How likely do you think you are to stop smoking if you would be diagnosed withcoronary heart disease in the next year?

- Extremely likely
- Somewhat likely
- Neither likely nor unlikely
- Somewhat unlikely
- Extremely unlikely

Have any of your blood-related family members (mother, father, siblings) been diagnosed with lung cancer?

- Yes
- No
- I don't know

Have any of your blood-related family members (mother, father, siblings) beendiagnosed with emphysema?

- Yes
- No
- I don't know

Have any of your blood-related family members (mother, father, siblings) been diagnosed with coronary heart disease?

- Yes
- No
- I don't know

Powered by Qualtrics
